# Insight into a Novel p53 Single Point Mutation (G389E) by Molecular Dynamics Simulations

**DOI:** 10.3390/ijms12010128

**Published:** 2010-12-30

**Authors:** Davide Pirolli, Cristiana Carelli Alinovi, Ettore Capoluongo, Maria Antonia Satta, Paola Concolino, Bruno Giardina, Maria Cristina De Rosa

**Affiliations:** 1 Istituto di Biochimica e Biochimica Clinica, Università Cattolica del Sacro Cuore, Largo F. Vito 1, 00168 Rome, Italy; E-Mails: davide.pirolli@rm.unicatt.it (D.P.); ecapoluongo@rm.unicatt.it (E.C.); paolaconcolino78@libero.it (P.C.); bgiardina@rm.unicatt.it (B.G.); 2 Dipartimento di Scienze Motorie e della Salute, Università di Cassino, Via S. Angelo-Località Folcara, 03043 Cassino (FR), Italy; E-Mail: cristiana.carellialinovi@rm.unicatt.it; 3 Istituto di Patologia Speciale Medica e Semeiotica Medica, Università Cattolica del Sacro Cuore, Largo F. Vito 1, 00168 Rome, Italy; E-Mail: msatta@rm.unicatt.it; 4 Istituto di Chimica del Riconoscimento Molecolare, Consiglio Nazionale delle Ricerche, Largo F. Vito 1, 00168 Rome, Italy

**Keywords:** p53, molecular dynamics, S100B, protein interactions

## Abstract

The majority of inactivating mutations of p53 reside in the central core DNA binding domain of the protein. In this computational study, we investigated the structural effects of a novel p53 mutation (G389E), identified in a patient with congenital adrenal hyperplasia, which is located within the extreme C-terminal domain (CTD) of p53, an unstructured, flexible region (residues 367–393) of major importance for the regulation of the protein. Based on the three-dimensional structure of a carboxyl-terminal peptide of p53 in complex with the S100B protein, which is involved in regulation of the tumor suppressor activity, a model of wild type (WT) and mutant extreme CTD was developed by molecular modeling and molecular dynamics simulation. It was found that the G389E amino acid replacement has negligible effects on free p53 in solution whereas it significantly affects the interactions of p53 with the S100B protein. The results suggest that the observed mutation may interfere with p53 transcription activation and provide useful information for site-directed mutagenesis experiments.

## 1. Introduction

Unstructured regions are a common motif of transcription factors [1]. One such example is the cancer suppressor protein p53, for which it has been outlined that unstructured and structured parts function synergistically [2]. p53 is a transcription factor that responds to oncogenic stress by inducing cell cycle arrest or apoptosis [3] and whose inactivation is mainly due to mutations that interfere with the DNA-binding activity of the protein [4,5]. The p53 protein consists of 393 residues and can be divided into three functional regions: (i) an N-terminal domain (1–93) containing a transcriptional activation domain and a proline-rich domain; (ii) a core DNA-binding domain (102–292), which contains most of the inactivating mutations found in human tumors; and (iii) a C-terminal domain (CTD) consisting of a tetramerization domain (320–356) and a regulatory domain (363–393). The extreme CTD, which binds to non-specific DNA sequences, is in fact of major importance for the regulation of the protein [6]. It seems to have a negative effect on specific DNA-binding activity of the core domain or by altering the conformation of p53 or by interfering by steric hindrance with the ability of the full-length protein to bind DNA [7,8]. Deletion of this regulatory region, binding of antibodies, phosphorylation and acetylation abolish the negative effect on DNA binding [9]. In particular, it has been observed that phosphorylation and/or mutations in this area enhance the stability of the protein [10–12].

Mutations of the tumor suppressor protein p53 are associated with more than 50% of human cancers; however, almost 30% of p53 mutations occur rarely and this has raised questions about their significance [13]. It therefore appeared of particular interest that we could identify a novel mutation (G389E) within the carboxy-terminal regulatory region, in a patient suffering from congenital adrenal hyperplasia (CAH) [14].

Computational methods have been successfully used to predict the effects of mutations on the structure and function of a protein before time consuming experiments are carried out [15,16]. In this study, investigations of structural consequences of this novel p53 G389E mutation are explored using molecular modeling and molecular dynamics (MD) simulations. In the absence of a three-dimensional structure of the whole extreme C-terminus, the NMR structure of a p53 peptide corresponding to Ser367–Glu388 residues, bound to S100B protein, was used as the starting structure for molecular modeling [17]. S100B, a member of the S100 family of EF-hand calcium-binding proteins, has been shown to contribute to cancer progression in malignant melanoma by interacting with p53 and inhibiting its function as a tumor suppressor [18]. As a result of S100B binding, important post-translational modifications on p53 are blocked and p53 dependent transcription activation is inhibited [19–21]. To provide an overall assessment of the effects of G389E mutation, MD simulations of wild type (WT) and mutant (G389E) peptides corresponding to the extreme CTD, free in solution and in complex with S100B, were then performed. Our results provide evidence to suggest that the novel mutation does not induce relevant structural changes of p53 CTD free in solution. Instead, analysis of MD trajectories highlights the potential effects of G389E replacement on the interactions of p53 with S100B.

## 2. Results and Discussion

### 2.1. Molecular Modeling

**Figure 1:**
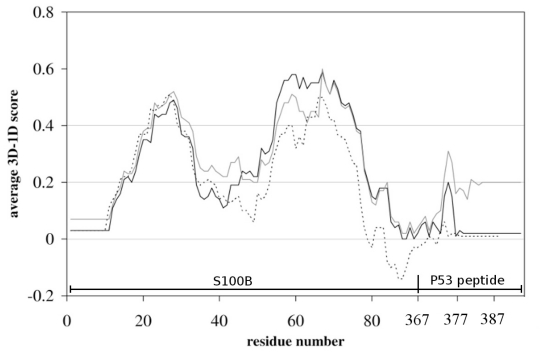
VERIFY-3D plots of molecular complexes of WT (*black continuous line*) and G389E (*grey line*) p53 peptide bound to S100B compared to the starting NMR structure 1DT7 (*black dashed line*). Although a negative score region is present in the template structure, the modeled structural motifs are characterized by the absence of negative score regions in the plots. Residues are numbered consecutively by VERIFY-3D from the amino terminus of S100B.

Twenty structural models of WT and G389E p53 extreme CTD were built using MODELLER [22] and the NMR complex between a p53 peptide (residues 367–388) and S100B as starting structure (pdb 1DT7) [17]. The 389–393 peptide sequence, which was not resolved in 1DT7 NMR structure, was modeled *ab initio* [22]. Among the obtained conformations of the generated complexes, one was selected on the basis of the lowest MODELLER objective function. The goodness of the selected folding was assessed by VERIFY-3D [23], which evaluates the compatibility of a given residue in a certain three-dimensional environment. A score below zero for a given residue means that the conformation adopted by that residue in the model is not compatible with its surrounding environment. As shown in Figure 1, the 3D-1D dimensional scores of the models generated by VERIFY-3D are always positive. The validity of the modeled structural motifs is highlighted by the absence of negative score regions in the plots; it is worth noticing that the VERIFY-3D plot of the NMR 1DT7 starting structure displays a negative score region (Figure 1).

The geometry of the selected models was also checked with PROCHECK [24] showing that no residue has a disallowed geometry.

### 2.2. Molecular Dynamics Simulations of WT and G389E Unbound Peptides

**Figure 2:**
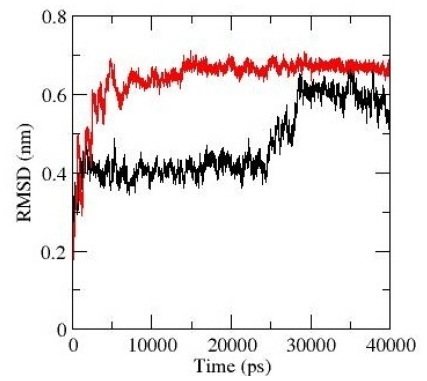
Backbone atom root-mean-square deviations (RMSD) for WT (*black*) and G389E (*red*) p53 extreme C-terminus domain free in solution from their corresponding starting structure.

The effects of G389E replacement on the unbound peptide conformations were evaluated by molecular (MD) simulations. The p53 extreme C-terminus exists as a random coil in its native form and becomes helical (residues Ser376-Thr387) when bound to S100B, as demonstrated by NMR spectroscopy [17]. Therefore, MD simulations at 300 K also enabled us to compare experimental data with the predictions drawn from computer simulated models determining whether the helix unfolds on a reasonable time scale. The simulations were started from the minimized structures of wild type and mutant peptides taken from the corresponding S100B model complexes and continued for 40 ns. Examination of backbone atom root-mean-square deviations (RMSD) (Figure 2) relative to the energy-minimized conformations, suggests that both peptides unfold (RMSD higher than 0.6 nm).

**Figure 3:**
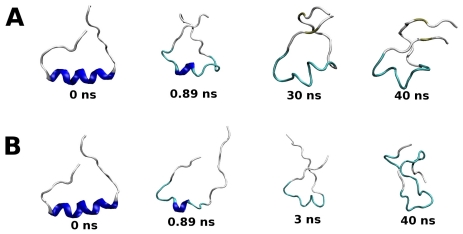
Representative snapshots taken from the 40 ns simulations of WT (**A**) and G389E (**B**) p53 extreme C-terminus domain free in solution.

Snapshots of the unfolding pathways are reported in Figure 3. The helix of the WT peptide rapidly unfolds by eight residues in the first 0.89 ns of simulation with an increase of the RMSD value to 0.41 nm (Figure 3A). The shorter helix spans residues 381–383 and it is stable to 25 ns, followed by further and complete unfolding at 30 ns (Figure 3A). In the simulation of the G389E peptide, the unfolding is more rapid and leads to higher RMSD values (Figure 2). A common feature with the WT simulation is the rapid loss of most of the helix structure in the first nanosecond (Figure 3B), but thereafter the RMSD increases rapidly reaching 0.6–0.65 nm in 3.0 ns when a complete loss of the helical structure was detected (Figure 3B). The snapshots of the mutant peptide clearly show a decrease in the time spent in the helical conformation compared to the WT peptide (Figure 3B). In agreement with the NMR studies, therefore, the MD simulations confirm that in the absence of S100B the p53 extreme C-terminus exists in solution in a disordered structure.

**Figure 4:**
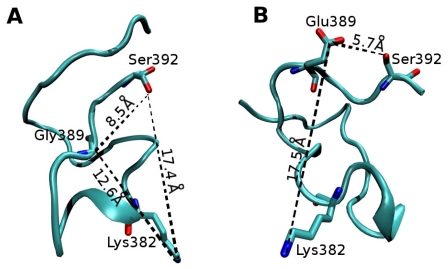
Cartoon representation of the snapshots of WT (**A**) and G389E (**B**) p53 peptide simulations at 40 ns showing the distances of residue 389 from the main phosphorylation and acetylation sites at the C-terminus of p53.

In addition, MD results show that replacement of Gly389 with Glu, occurring in a random coil region, causes localized structural changes with no significant effects on the phosphorylation and acetylation sites near the carboxyl terminus. An illustration of the snapshots of WT and G389E p53 peptide simulations at 40 ns is reported in Figure 4.

The figure displays the distances of Glu389 residue from Lys382 and Ser392, and highlights the negligible effect exerted by position 389 on the main acetylation and phosphorylation sites at the C-terminus of p53 [6]. Analysis of MD trajectories indicates that during the entire 40 ns of simulation, Glu389 is solvent exposed and does not interact with other residues of the extreme CTD.

### 2.3. Molecular Dynamics Simulations of WT and G389E Peptides Bound to S100B

The available NMR structure of p53 in complex with S100B gave us the opportunity to assess the role of this mutation also in the context of the intermolecular interactions which down-regulate the tumor suppressor function. The interaction of p53 with S100B is of particular interest because while most proteins that bind or modify the C-terminal of p53 activate the tumor suppressor, the opposite effect is observed for S100B which causes inhibition of phosphorylation and of p53 dependent transcription activation [20,21]. It was found that phosphorylation (at positions Ser376, Thr377) and acetylation (at position Lys382) reduce the affinity of the S100B-p53 interaction [25] and are therefore important for protecting p53 from S100B-dependent downregulation.

A 40 ns classical MD simulation including explicit water of the two complexes was carried out.

**Figure 5:**
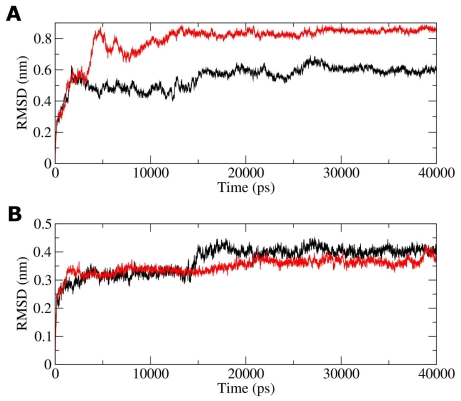
RMSD plots of S100B complexes throughout the MD simulations. (**A**) Backbone atom RMSD with WT (*black*) and G389E (*red*) peptide of p53 included; (**B**) Backbone atom RMSD with WT (*black*) and G389E (*red*) peptide of p53 excluded.

The root mean square deviation (RMSD) of the backbone atoms relative to the corresponding starting structures was calculated and the results are reported in Figure 5.

As shown in figure, both complexes are rather flexible (Figure 5A). A closer inspection of the RMSD indicates that its variation is mainly due to the C-terminal p53 peptide, this effect being more pronounced in the G389E complex, while S100B is rather stable in both simulations (Figure 5B).

**Figure 6:**
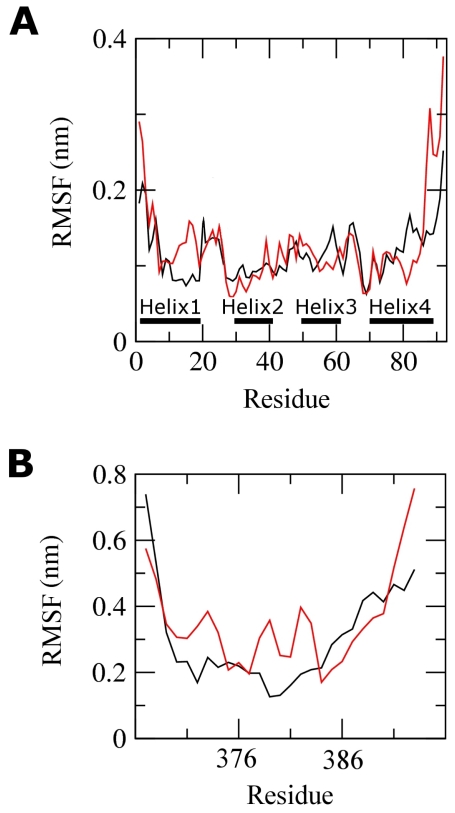
Residue-based Cα RMSF relative to the average structure for S100B (**A**) and p53 peptide (**B**) in WT (*black*) and G389E (*red*) complexes.

The deviations described above indicate how far the protein has moved from the starting structure. The Cα root-mean-square fluctuations (RMSF) with respect to the average MD conformation are extremely appropriate to describe flexibility differences among residues (Figure 6).

The RMSF profile over the whole simulation period of 40 ns shows that comparable fluctuations were found for S100B in both WT and G389E runs (Figure 6A). Large fluctuations from the averaged MD conformations were found at the C- and N-termini while the four S100B helix structures (2–19, 29–40, 49–61 and 70–88) were consistently stable, with RMSF around 0.1–0.15 nm. A detailed analysis reveals a higher flexibility in few turns of helix 3 and helix 4 of the WT complex compared with the mutant one. A larger fluctuation of helix 1 (residues 2–19) was observed in the G389E run. Taken together, the substitution of Gly389 in p53 does not destabilize the overall organization of S100B.

Consistent with the RMSD analysis, the G389E peptide also displayed a larger RMSF as compared to the WT (Figure 6B). A remarkable enhancement in mobility of the helix segment of the peptide is observed, which can be thought of as an adaptive motion.

Secondary structure analysis was carried out to explore whether the sizable RMSD and RMSF of the p53 peptide result from a different orientation in the MD simulation compared to the starting structure or from secondary structure changes.

**Figure 7:**
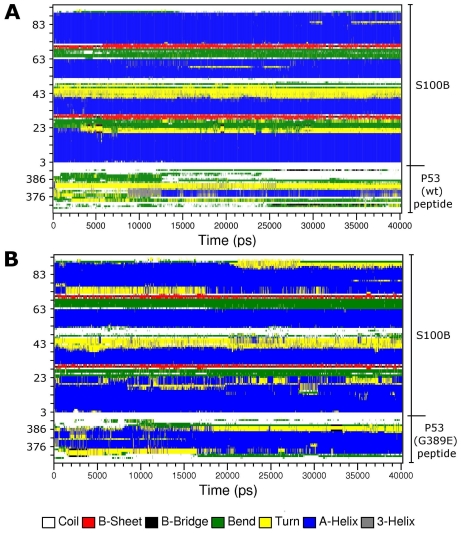
Secondary structure of S100B complexes as a function of time. Results for WT peptide- and G389E peptide-S100B complexes are reported in (**A**) and (**B**), respectively.

According to NMR studies [17], much of the C-terminal p53 peptide (Ser376–Thr387) adopts a helical conformation when bound to the S100B protein. The binding surface in the p53 peptide-S100B complex is defined by few residues from loop 2 (extending from residue 41 to 48), helix 3 (extending from residue 49 to 61) and helix 4 (extending from residues 70 to 87) of S100B and by residues 375–388 of p53. The time evolution of the secondary structure elements is depicted in Figure 7.

In the WT, run all secondary structure elements of S100B can be considered as stable. The peptide helical conformation (residues 376 to 387) is lost during the simulation and partially refolds into a sort of helix, which is assigned as an α-helix and transiently as a 3_10_-helix (Figure 7A). In the case of the G389E run, we observe that S100B helix 4 is less persistent, with a loss of α-helix at its terminus, whereas the mutant peptide increases its α-helix content (Figure 7B).

**Figure 8:**
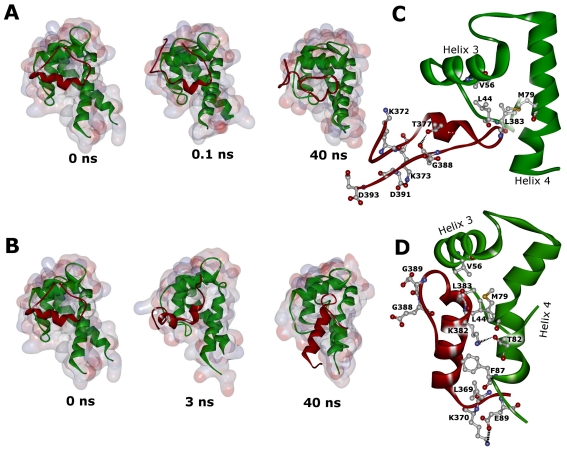
Snapshots from the simulated complexes during the MD runs. Plots of WT (**A**) and G389E (**B**) complex intermediates generated by the MD simulation at the indicated times. S100B and p53 peptides are represented by green and red cartoons, respectively. The loss of secondary structure in the WT peptide (**A**) and the movement of the helix in the G389E peptide (**B**), as a function of time, are noteworthy. Average structures over the last 5 ns of simulation of the WT and G389E complexes are reported in panel (**C**) and (**D**), respectively.

In Figure 8 snapshots of the 40 ns MD simulations for the two complexes are presented.

Focusing on the WT p53 peptide, during the first 0.1 ns, the helical structure starts to unfold (Figure 8A). The instability of the p53 C-terminal peptide in complex with S100B during MD simulation has been already reported by Whitlow *et al.* [26]. It is worthwhile noting that they used two different force fields, namely CHARMM27 and AMBER, and their findings are in excellent agreement with ours, *i.e.,* the WT complex is not stable in the MD with a backbone RMSD comprised between 5 Å and 6 Å in the last 5 ns of simulation, values comparable with those found in Figure 5A. They also observed, in agreement with our study, that the helical structure of the p53 C-terminal peptide is gradually lost during simulation. A possible explanation for this result may be that the C-terminal peptide is only a small fraction of p53 and therefore characterized by structural instability [26]. At the end of the simulation, a large conformational change of the WT p53 peptide is observed, coinciding with a severe loss of α-helical structure (Figures 8A and 7A). This conformational change starts after 28 ns of the MD simulation. Snapshots from simulation show that the WT peptide is in a closed conformation. Favorable electrostatic interactions between the charged residues within the p53 extreme CTD enable bending of peptide in correspondence with the mutation site. The residues responsible for these electrostatic interactions were the negatively charged Asp391 and Asp393, which alternatively interacted with positively charged Lys370, Lys372 and Lys373. Direct salt bridges have been assumed to be around 4.3 Å, whereas indirect or water-mediated salt bridges have been assumed to have a distance between 4.3 and 7.0 Å [27]. The direct salt bridge between Asp391 and Lys373 was very strong (Figure 8C) persisting through most of the MD simulation. A hydrogen bond between the O backbone atom of Glu388 and the OG1 atom of Thr377 is indicated by a distance of 2.6 Å in the average structure, contributing to the closed peptide conformation. This interaction is stable during most of the MD simulation based on a critical hydrogen bond distance of <3.5 Å.

Replacement of Gly by Glu, which is expected to restrict the conformational freedom of the peptide backbone at this point, imparts a more rigid structure to the corresponding region of the p53 C-terminus and prevents the p53 extreme CTD from its bending (Figure 8B). Moreover, introduction of the negatively charged Glu389 residue moves Glu388 far away from potential interacting C-terminal residues. This results in a different orientation of the peptide and in a slight increase of its helical content as the simulation progresses (Figures 8B and 7B). Both effects may therefore account for the increased RMSD observed for the mutant p53 peptide (see Figure 5A). First, only few turns of the peptide helix remain packed against helix 4 of S100B, and thereafter, the whole peptide helix is flanking helix 4 (Figure 8B). In the mutant complex, the salt bridges observed within the WT extreme CTD were never formed, while Lys382 and Lys370 stably anchor the mutant peptide to helix 4 of S100B. In fact, the distance between Lys382 NZ atom and Thr82 OG1 is below the hydrogen bond distance threshold (Figure 8D) as well as the distance between Lys370 NZ atom and Glu89 OE1 atom becomes stable during the MD run forming a direct salt bridge (Figure 8D).

In both WT and mutant systems the hydrophobic Leu383 of the p53 C-terminal interacts with a hydrophobic patch on S100B involving Met79, Leu44 and Val56 of S100B (Figure 8C, D). Upon G389E mutation, and as a consequence of peptide moving, the hydrophobic residue Leu369 provides favorable contributions to hydrophobic force establishing a close contact with S100B Phe87.

On the basis of MD results, we therefore predict that the stability of the p53 peptide-S100B complex, upon G389E substitution, is increased. As a consequence, the affinity of the S100B-p53 interaction might be altered and the biological function of p53 as a tumor suppressor further disabled.

## 3. Experimental Section

### 3.1. Molecular Modeling

The sequence of the extreme C-terminus (residues 367-393) of p53 is

SHLKSKKGQSTSRHKKLMFKTE**G**PDSD

with the amino acid 389 in bold.

The three-dimensional structure of the carboxyl-terminal peptide of p53 (residues 367–388) in complex with S100B protein was taken from the Protein Data Bank (code 1DT7 [17]) and used as initial structure for molecular modeling. The sequence 389–393, which was not resolved in 1DT7 structure, was built with an *ab initio* modeling routine of MODELLER as implemented in Discovery Studio (Accelrys Inc.) [2]. Twenty alternative conformations of the WT and G389E extreme CTD in complex with S100B were generated. The geometrical consistency of the models was evaluated based on the probability density function (PDF) violations provided by MODELLER. After visual inspection using the computer graphics program Discovery Studio 2.1 (Accelrys Inc.), a representative model from each set, used for all subsequent studies, was chosen, which corresponded to low restraints violations and to favorable geometrical and stereochemical properties as checked with VERIFY-3D and PROCHECK [24,28].

### 3.2. Molecular Dynamics Simulations

Forty nanosecond MD simulations of WT and G389E extreme CTD, free in solution and in complex with S100B, were performed using GROMACS software version 4.0.5 with the OPLS-(AA)/L all-atom force field [29]. For the simulations of unbound peptides, the starting structures were extracted from the complexes with S100B. Each peptide and complex was immersed in a cubic box extending to at least 0.9 nm from the protein surface and solvated with explicit SPC water molecules. Chloride and sodium ions were added to neutralize the systems (peptides and complexes, respectively) which were then simulated with periodic boundary conditions. The final peptide systems consisted of 435 and 443 protein atoms (wild type and mutant, respectively) surrounded by ~4,000 water molecules, whereas the final complex systems consisted of 1,895 and 1,903 protein atoms (wild type and mutant, respectively), surrounded by ~13,000 water molecules. Before running the simulation, each system was energy minimized for 1,000 iterations of steepest descents and then equilibrated for 20 ps, during which the protein atoms were restrained. All restraints were then removed from the complexes and the temperature was gradually increased in 10 distinct steps of 5 ps simulations each.

Berendsen coupling was employed to maintain a constant temperature of 300 K with a coupling constant τ of 0.1 ps. van der Waals interactions were modeled using 6–12 Lennard-Jones potentials with a 1.4 nm cutoff. Long-range electrostatic interactions were calculated using particle mesh Ewald method [30], with a cutoff for the real space term of 1.2 nm. Covalent bonds were constrained using the Lincs algorithm [31]. The time step employed was 2 fs and the coordinates were saved every 5 ps for analysis of MD trajectories. Secondary structure was calculated using the DSSP algorithm [32] within GROMACS and all other analyses were performed using GROMACS utilities.

## 4. Conclusions

In this study, the main goal of MD simulation was essentially to make predictions of the effects of a novel p53 mutation (G389E), opening possibilities for exploring properties of the molecular system under investigation that are less accessible by conventional experimental methods. Our main conclusions are as follows. First, our studies indicate that the p53 G389E mutation has negligible effect on the protein when it is free in solution. Second, (in agreement with previous simulations of the p53 extreme C-terminus in complex with S100B [26]) the helical WT peptide gradually loses its secondary structure during simulation. Third, and perhaps most importantly, it was found that in the presence of this novel mutation the binding surface between the p53 C-terminus and S100B is significantly altered, thus further preventing p53 from functioning. The results reported herein strengthen the role of the intermolecular interactions in p53 activity and provide useful information for the design of appropriate mutants.
